# Nursery Government in Its Sanitary Aspects

**Published:** 1857-07

**Authors:** T. Herbert Barker


### NURSERY GOVERNMENT IN ITS SANITARY ASPECTS.

By T. HERBERT BARKER, M.D., F.R.C.S.

(*Continued from* vol. ii, p. 373.)

Clothing of Children. i. *General Observations.* In every period of life, health depends, in a great measure, on the equable diffusion of warmth over the whole body; and this cannot be insured, in our variable climate, without special care in clothing. Yet it is possible that some reader, pointing to the children of the poor, thinly clad, and often rudely exposed to the weather, may ask, "Are not some of those who are most exposed robust and healthy?" I reply, that such exceptions, when duly considered, will only serve to confirm the rule I have laid down. It is necessary, however, to refer to these cases, because on a few supposed instances of the beneficial effects of exposure, some persons have grounded an absurd and mischievous theory of "hardening" children. To meet these theorists with facts, I refer to the register of deaths, where it is found that, of children under seven years of age, there is a much greater mortality among the poor than among the rich. Although I am aware that there are other fruitful sources of mortality among the children of the poor, it cannot, I think, be reasonably doubted that cold, or the want of suitable clothing and shelter, adds greatly to the number of early deaths.

But a reference to the provisions made by nature for the warmth of young animals will best serve to refute the advocates of the "hardening" system. Let us notice the covering of soft warm down on the chicken (which preserves heat better than the feathers which are afterwards developed), or observe the hair on a young foal, which is both softer and warmer than that of the full-grown animal; and from these and numerous similar circumstances, we may learn that the rule of nature is to take especial care of the temperature of young animals. If the study of clothing, then, is generally important, it is especially so with regard to the health of children. In my remarks on clothing and temperature, I would recommend a middle course between the two extremes to which many infant lives have been sacrificed—the "hardening" process on the one hand, and the dread of fresh air on the other.

As nature strengthens the child, we may second her efforts by exposing it to such moderate degrees of cold as its constitution is well able to resist; but we must by no means expect that a child, naturally feeble, will be hardened and invigorated by a want of sufficient clothing, or a pitiless exposure to extreme cold. It is obvious that a young and feeble child must require more clothing than one older and stronger, who can take vigorous exercise.

Our observations on this point are confirmed by a reference to the statements of able physiologists. Dr. Edwards has shown by experiments that the natural heat of mature infants at birth is from 3° to 5° below that of adults (the heat of premature infants is still lower), and that the power of producing heat is at its *minimum* at birth. The practical deductions from these facts are obvious—that the newly born infant should be warmly wrapped in flannel; that during cold weather it should be dressed near a comfortable fire; and that all other proper means should be used to preserve a due degree of warmth. "Instinct," says Dr. Edwards, "leads mothers to keep their infants *warm*, though philosophers, by more or less specious reasoning, have at different times and in different countries induced them to abandon this guide, by persuading them that external cold would fortify the constitutions of their children as it does those of adults."* The same writer, in treating of mortality from *cold*, observes—"It is not confined to children whom the misery of their parents cannot guard from the rigour of the weather, but it also operates, to a great extent (without being either perceived or suspected), in families enjoying affluence, and where it is believed that the necessary precautions have been taken; because *cold*, being relative, it is difficult from our own feelings to judge of its effect on others, and because it does not always manifest itself by determinate and uniform sensations. They do not feel the cold, but they have an uneasiness or indisposition arising from it; their constitution becomes deteriorated by passing through the alternations of health and disease, and they sink under the action of an unknown cause. It is the more likely to be unknown, because the injurious effects of cold do not always manifest themselves during or immediately after its application. The changes are at first insensible; they increase by the repetition of the impression, or by its long duration, and the constitution is altered without the effect being suspected."

For further proof that it is not the children of the poor

* Dr. Edwards on the Influence of Physical Agents on Life. Translated by Drs. Hodgkin and Fisher.

only who suffer from cold, we may quote the remarks of Drs. Evanson and Maunsell: "We wish," say they, "we could adequately depict one of those miserable victims of parental vanity whose appearance in our streets will sometimes, on a March or November day, strike cold into our hearts. The cap and feathers set upon (not covering) the child's head, and probably of a colour and richness contrasting mournfully with the blue ears, sharpened nose, and shrunken cheeks (in which cold has assumed the features of starvation); the short kilt and Highland hose, exposing between them cracked and quivering knees, altogether require for their description more graphic power than we can presume to lay claim to."*

As a fact worthy of notice in this place, I may quote a well authenticated statement that, during cold winters in Italy, it has been observed that many deaths of infants resulted from the practice of taking them to be baptised in cold churches; as soon as the clergy put in force their new orders to baptise young children at home during severe weather, the amount of infant mortality was greatly abated.

MM. Villermé and Milne-Edwards have shown that both extreme heat and cold are highly injurious to the infant, and that the mortality among infants is considerably increased during the months of January, February, and March, when we commonly have the most inclement weather.

The statements of these distinguished scientific men are confirmed by Dr. Caffort, of Narbonne, whose tables of mortality of children, in Narbonne, show the following figures:—Average mortality, 1 in 9·57; in January, 1 in 9; in April, 1 in 10; in May, 1 in 17; and in June and July, from 1 in 7 to 1 in 8. Still more convincing facts are observed in Russia, where the number of deaths of infants increases rapidly from the more temperate to the severer latitudes. In Russia—taken as a whole—the mortality among children exceeds that among persons of all other ages taken collectively; and, as we advance from the mild temperature of Courland to the rigorous climate of Tobolsk, we find, at the latter place, the ratio of deaths *almost doubled*. These are facts—and many others might be added—which demonstrate the cruel absurdity of the so-called "hardening system," which exposes tender infants to extreme cold.

* A Practical Treatise on the Management and Diseases of Children. By Drs. Evanson and Maunsell.

I must return to this topic, deficient clothing, in my special remarks ; at present, I have to consider another very serious evil, and one far too common—the habit, namely, of wearing certain parts of clothing so *tight* as to interfere with the circulation of the blood and the development of the muscular system. Of this evil the most injurious instance is found in *tight-laced stays,* as worn by young ladies ; but some other cases also require notice. Even infants are frequently found, suffering simply from the effects of tight clothing, while their indications of pain are referred to other causes. I have been called to attend infants, apparently labouring under general indisposition, with restlessness, embarrassed breathing, sickness, and a dusky hue of the skin, in place of the rosiness of health, and all this the effect of a too tight application of the band, or of some ill fitting article of dress; and, greatly to the surprise of both mother and nurse, a cure has been soon effected in such cases, simply by loosening the dress, without any medicinal administration. Tight-waisted trowsers for boys, and stays of all kinds for girls, should be interdicted during the whole period of childhood, nay, of life. But the evils of *stays* are of the most serious character. Besides crippling muscular action, their continued pressure alters the shape of the chest, diminishes the capacity of the lungs for air, forces the stomach, liver, and spleen, from their natural positions, presses these upon each other, and diminishes their functional power. As an inevitable consequence, this produces derangement of the respiration, the circulation, and the digestion, and too often leads to an early grave, or to a life of prolonged ill health. All these effects, too clearly and too frequently demonstrated in the *post mortem* science, surely indicate penalties too dear to pay for the sake of complying with the absurdities and cruelties of fashion. We scoff at the Chinese for crippling the feet of their ladies; while a still more unreasonable custom prevails among ourselves. If "fashion" *must* be allowed to do violence to some part of the human frame, then by all means let the feet be given into her service, not the heart and the lungs. "It is generally thought that stays improve the shape. There cannot be a greater mistake."* We shall conclude our remarks on this topic with a quotation from the *Report of the Registrar-General of Births, Deaths, and Marriages:*—

"The higher mortality of Englishwomen by consumption

---

* Coulson, On the Injurious Effects of Tight Lacing.

may be ascribed partly to the in-door life which they lead, and partly to the compression, preventing expansion of the chest, by costume. In both ways they are deprived of free draughts of vital air, and the altered blood deposits tuberculous blood with fatal and unnatural facility. *Thirty-one thousand and ninety Englishwomen died in one year* (the year ending June 30, 1839) of this incurable malady! Will not this impressive fact induce persons of rank and influence to set their countrywomen right in the article of dress, and lead them to abandon a practice which disfigures the body, strangles the chest, produces nervous and other disorders, and has an unquestionable tendency to implant an incurable hectic malady in the frame? Girls have no more need of artificial bones and bandages than boys."

II. *Special Remarks on Clothing for the Head, Chest, Abdomen, and Limbs.* As a general rule, we may observe that the *head* should be kept cool. It is so well protected by nature, that, excepting to guard it from draughts and extreme cold, it requires but little care. Yet we sometimes find the head jealously guarded from cold, while the parts of the body more susceptible of injury—the chest and the feet—are left with insufficient protection. The *chest* and the *abdomen* should be kept warm, in order that the blood may not be repelled from the surface, and driven in undue proportions upon the internal organs. While the infant is unable to take that exercise which in after life serves to maintain a vigorous circulation, it is highly important that the body should be thoroughly protected from cold and changes of temperature. For this purpose, no sort of clothing is so suitable as Welsh flannel of the finest and softest quality. The infant cannot be dressed better than in flannel sufficiently long to fall some nine or ten inches below the feet, for the first four or five months. After this period, when the child is able to exercise its limbs, and it is desirable to shorten the clothes, woollen socks or stockings should be substituted.

When the child is older, and can take exercise in the fresh air, the continued use of flannel next the skin has this great advantage, that the fresh air can be enjoyed more freely in various weather, without risk of taking cold. In cold weather, woollen gaiters should be drawn over the legs when the child is exposed to the open air. No other clothing can be a sufficient substitute for flannel; but we may remark here, that cotton, being considerably warmer than linen, is more suitable for children.

Here a remark may be added respecting change of garments. The extra clothing in which exercise has been taken should not be immediately and altogether removed while the child is very warm or perspiring. Much of the disrepute into which warm clothing has been brought by some, may be attributable to the mischief which undoubtedly arises from abrupt changes of dress. The limbs should be free from all bandages and ligatures, and the whole clothing should be so loose that there can be no restraint on the movements. It is advisable, too, that the articles of infant dress should be tied rather than pinned. Attention to this suggestion will probably prevent much discomfort, and even danger.

The custom of exposing the whole of the neck and the upper part of the chest in the young female ought to be severely reprehended. It cannot be practised without serious risk in our changeable climate, and especially when other habits tend rather to make the body susceptible of cold than otherwise. Boys should wear warm double-breasted waist-coats in winter; this is preferable to the use of hair-skin. Respecting night-dresses we may observe that, in the winter, if children have the habit of throwing the bedclothes from them, a flannel night-gown may be used; in other cases, one of calico will be sufficient.

The trousers worn by little girls, and by boys before they are breeched, are too commonly made of very flimsy materials, affording hardly any warmth. Flannel or jean should be used for these parts of clothing.

The *feet* require especial care to preserve them from cold and damp. When the extremities—the hands and feet—are habitually allowed to suffer from cold, the circulation is necessarily unequal, and there must be some risk of producing injurious effects on some of the internal organs, particularly the brain, the lungs, and the bowels. I have repeatedly observed the happy effects of careful attention to the warmth of the extremities, in relieving the embarrassed functions of the internal organs. In the winter season, the shoes should be thick and perfectly waterproof. When a youth is delicate, double soles, with a layer of cork or bladder between them, are advisable.

Wet feet are a most frequent source of cold; therefore, when, on returning from exercise, the feet are in any degree damp, the shoes and stockings should be immediately changed. Soles of *gutta-percha*, or overshoes made of India-rubber, may be recommended here, as excellently adapted, not only to keep the feet dry when the ground is wet (as after a thaw of frost or snow), but also to fence the foot against the cold in the most secure mode possible. Care should be taken that all shoes and boots are made easy to the feet. Corns, bunions, etc., which are almost always the effects of tight or ill fitting shoes, may be avoided by early attention to the comfort of the feet. Shoes must be preferred to boots, as the latter, especially when made with stiff upper leathers, impede the motions, and consequently confine the symmetrical growth of the ankle-joints and other parts.

Temperature. In the section on clothing, I have anticipated some of the practical remarks which might have been given under the present title; but a few additional observations may serve to confirm the opinions already stated regarding the effects of warmth and cold.

In the first place, I may again notice a popular fallacy to which reference has been made in my remarks on the "hardening" system of training children. I allude to the belief that exposure to heat always renders the body more susceptible of the ill effects of cold. There is some slight bsais of fact in this common opinion; but it is by no means true in the unrestricted sense in which it is often understood, while the inferences drawn from it are both incorrect and mischievous.

To clear up this matter, I will first give an instance of a fact which seems to support the common notion. A boy, after engaging in violent exercise, so as to produce free perspiration, sits down to rest in a current of cold air, and remains there until his body is chilled; the consequence doubtless is, that he suffers from that obstruction of perspiration which we term "a cold". Now, observe, the mischief here arises, not from the fact of a sudden change of temperature (as in passing from a warm conservatory into the cool open air), but from the fact that the body is exposed to a greater degree of cold than it is able to resist. It by no means follows that the boy would have suffered in the same way by leaving a warm parlour and walking a mile or more exposed to the keen frosty air of a winter's morning. Why? Because, by the store of warmth accumulated in his system while he sat by the parlour fire, he is now well able to resist the degree of cold to which he is exposed.

I might easily support this doctrine by reference to numerous undoubted facts. One is too conclusive to be passed over. If any people ought to know the proper means of resisting cold, it must be the Russians, and other inhabitants of the frozen North. Now, the Russians and other natives of northern countries preserve, by means of stoves and double doors and windows, a very high degree of heat in their houses during winter, and yet leave them with impunity to pursue their occupations in the open air. One of their common practices is to leave the vapour bath, and, while in a glowing and steaming condition of heat, to roll themselves in the snow.* Dr. Edwards also found, experimentally, that "in exposing animals to successive applications of cold, their temperature fell the more slowly the longer they had been subjected to the influence of warmth." From this he deduces the obvious consequence—"that those who are liable to frequent exposure to severe cold, are rendered more capable of supporting it by subjecting themselves in the intervals to a high temperature."

The nursery should not be kept at an immoderately high temperature. In winter, the heat should not exceed 60° Fahrenheit. A *good* thermometer (this precaution is necessary, for the instruments commonly sold are often found to be worthless) should be considered an indispensable appendage to the nursery. In recommending the use of a *thermometer*, I do not intend to imply that all its minute variations must be attended to, so as to lead us to alter the dress or abridge the open air exercise of children on account of slight changes of temperature; but this simple instrument might with great advantage be more generally used to indicate the more important, or the wider and more abrupt, changes from heat to cold, and *vice versâ*, to which our climate is so liable. It is not generally understood that the temperature of morning and evening in spring differs in an important degree from that of the same hours of the day in autumn. In April, the mornings and evenings are cold, because, in the absence of the sun, the damp and cold soil speedily reduces the temperature which the air has acquired during the day. In October, the mornings and evenings are comparatively warm, because the soil, warmed by the summer months, does not so soon depress the temperature of the air. This is one of the facts indicated by the thermometer, and is evidently one of some practical interest to delicate persons, and those who have the care of children.

I am so far a believer in the injurious effects of the *east wind*, that, during its prevalence, I would recommend especial

* "The common winter temperature of houses in St. Petersburgh is 64° Fahrenheit; whereas, out of doors, it is frequently as low as — 20°." (Dr. Glanville's Travels.) For many useful observations on temperature, the reader may consult a valuable little essay, entitled "Thermal Comfort", by Sir George Lefevre, M.D.

care to be taken with regard to temperature and exposure to cold. If there is in our climate any time (excepting heavy rains or snow storms) when we would intermit daily exercise in the open air, it is when a keen easterly wind is blowing. I am inclined to think that, after all the numerous observations which have been made upon it, there is still something in the east wind which has not yet been sufficiently explained. Its peculiar, benumbing, and deadening influence upon animals, and especially upon *fish*,* is very remarkable. Some of its effects, however, may be at least partly explained. The peculiar chillness which it produces, so annoying to persons of a rheumatic habit, may, in a great measure, be traced to its extreme dryness. This causes rapid evaporation from the skin, and consequent cold. The dew-point in spring, and during an easterly wind, is frequently many degrees below the temperature of the air. The effect of such a state of dryness is parching in the extreme to the skin of animals, as well as to the leaves and blossoms of plants. The low dew-point permits the temperature to sink greatly below that of the day, and this increases the injurious nature of the night air in spring.

Such sudden and considerable changes seem to require modifications of our bed-clothing during the night, as well as of our body-clothing during the day. Yet, the general mode is to have one fixed quantity of covering for the bed in summer and another for the winter, while we neglect other and more abrupt changes. In the case of children, the injury likely to result from such transitions may, in a great measure, be prevented by the use of suitable night-dresses, made *long;* of linen in summer, but of calico in winter. When the nights are very cold, young children may be still further protected by a light warm shawl crossed over the chest, but not so tightly as to interfere in the least degree with respiration. I may add that, if linen bed-clothing be used in summer, it will be well to substitute calico for the winter.

Air. As the important influence of fresh air, or ventilation, in the management of infancy, claims some further notice, beyond the incidental allusions to it already made, I propose to give a few concise observations upon it in a distinct section.

---

* It is a fact well known by all experienced anglers, that during an east wind, even when the weather is not severely cold, fish generally refuse to take a bait. The *perch*, styled a "bold" fish, and the voracious *pike*, seem to have lost their appetite at such a time. There may be exceptions; but such has been the rule observed ever since the days of Izaac Walton, who prays "that the east wind may not blow" when his disciple goes "a fishing."

The most obvious means of securing a good ventilation (which must be understood as quite distinct from any thing like a violent and chilling draught of air) are, first, a sufficient number of windows for the size of the room; and second, an open chimney. Where these simple means are found not sufficient to produce a free ventilation, we may recommend the excellent "ventilator" invented by Dr. Arnott, which may be fixed in any room at a slight cost, and has, we believe, no accompanying disadvantages. It is obvious that a large room has its advantages; that the windows of a nursery should be thrown open when the children are not there, and, in fine weather, should be frequently partially opened when the children are there. A change of rooms is very desirable, when children are long confined to the house from inclemency of weather. In short, let it be understood well, that a supply of pure air is as much needed by the lungs of the child as the supply of wholesome food is required by the stomach, with this important difference—that, while in the latter instance, the demand is only occasional, in the former it is perpetual. Gymnastic exercises in the nursery are useful; but they must never be regarded as a sufficient substitute for the natural play of children in the open air.

Sleep. It is one part of the beautiful arrangements of nature, respecting the infant, that it should be *gradually* introduced to the activity and excitement of life. Accordingly, we find that the early period of infancy is passed in *sleep*, and that (apart from disease or uneasiness, resulting from circumstances,) the only natural interruption to repose is the feeling of hunger. The lesson to be deduced from this fact is obvious,—that all unnecessary interruptions of the child's natural state of rest should be avoided.

When a child shows sleeplessness, we have reason to suspect some disorder of the stomach or bowels. When the latter are sufficiently open, the motions are of a healthy bright yellow colour, and free from *mucus*. Flatulency will often be found to be the cause of want of sleep; and, in this case, an occasional dose of rhubarb and magnesia, or of castor-oil, will generally prove the most suitable kind of composing medicine.* I, of course, suppose that every judicious mother will ask the advice of her medical attendant before she employs the dangerous powers of any *opiate* preparations, for the purpose of procuring sleep for the child, and quiet for herself.

* Twinberrow's lemon-peel-flavoured castor-oil is an excellent preparation for infants and children.

Leaving the period of early infancy, I must say a few words on the great importance of training the child to regular habits in retiring to rest. Six o'clock in the winter, and seven in the summer, are suitable hours, and nothing should be allowed to interfere with the regular time allotted for sleep. Children who are frequently allowed to stay up late (as a treat!) become feverish, excitable, and more and more indisposed to retire to rest at the proper time. With regard to the amount of sleep to be allowed to a child, no exact rule can be laid down—for one child may require more than another; but when a child is trained to go to bed soon after its last evening meal, and is permitted to sleep without disturbance, and wake of its own accord, then, if the waking hour is found to be too late, we may conclude that the hour of retiring to rest has not been sufficiently early. Early rising cannot be successfully inculcated without strict attention to regularity in going to bed. During the period of boyhood, (or from eight to fifteen years of age,) the hour of retiring to rest should not be later than *nine.* We may then reasonably expect the boy to rise, perfectly refreshed and invigorated, at *six* in the morning. Let it be remembered, that not merely physical health, but character and success in life, may depend on the inculcation of these regular habits.

The material employed for the beds of children is of some importance. It should be firm, somewhat elastic, and such as may be readily dried. A small tick, filled with straw or oat-chaff, will be suitable for young children; but the straw or chaff should be frequently changed. When the children are older, a hair mattress may be substituted. This should be exposed daily to a free current of air, and in summer it may be well to place it sometimes out of doors, in the sunshine for an hour or two. The luxury of a soft feather bed cannot be in any way recommended as suitable for children. Nor can we recommend night-caps. Care being taken that the head is not exposed to a draught of air, a child may safely and advantageously sleep without the incumbrance of a cap. The notion that sleeping without a night-cap is injurious to the hair, is quite erroneous. Excepting the cases of infants sleeping with their mothers and nurses, it is not advisable that young children should sleep with adults, and it is especially injurious when they sleep with elderly persons, as Dr. Roget has clearly proved that, in this case, heat is unduly abstracted from the infant body.

BATHING, ETC. *Washing.* Too much attention can hardly be paid to cleanliness during infancy and childhood. Careful washing should be performed daily, until the child is able to attend to its own comfort. In the early stage of infancy, warm water may be used with a fine sponge, and care should be taken not to allow exposure of the body so long as to produce fatigue or any severe impression of cold. We have known bad results to follow too protracted exposure in washing, and tardy movements in dressing the child. These are errors particularly likely to be committed by an inexperienced mother, for the first time taking charge of an infant.

As the child grows stronger, and when the weather is warm, we may gradually diminish the temperature of the water, until we may use it nearly or quite cold; but there should be no haste in employing very cold water. It will generally be well for the water which is to be used in the morning, to remain in the nursery during the night, in order that it may acquire the temperature of the air of the room. I strongly protest against the common error of bathing *infants* in cold water with a view to "hardening" them; they should have daily tepid ablutions only, unless in summer weather, when the water is comparatively warm.

*Bathing*. Perfect cleanliness cannot be secured without the frequent immersion of the body by bathing, and less than this will not suffice to keep the skin in a healthy and perspirable condition; indeed, without frequent bathing that healthy vigorous action of the vessels of the skin cannot be maintained, which is so conducive to health in the child as well as in the adult. The now frequent use of the bath in its various forms, is one of the few continental customs which we might adopt with advantage; but it is gratifying to observe, that public attention in this country is increasingly directed to this cleanly and wholesome habit.

As far as it relates to the management of infancy and childhood, I will give a few concise directions on the subject of bathing.

Baths are either *hot*, *warm*, *tepid*, or *cold*.

| | | | | |
|---|---|---|---|---|
| The temperature of the *hot* bath | is above | .... | 98° Fahr. | |
| ,, ,, *warm* | from | .... | 85° to 98° | |
| ,, ,, *tepid* | ,, | .... | 65° | 85° |
| ,, ,, *cold* | ,, | .... | 32° | 65° |

The temperature of the morning and evening bath should be about 90° or 96° Fahr.; and on no account should the infant be submitted to *cold* bathing during the first few weeks of its existence.

"To use cold water," writes Dr. Conquest, "at this early stage of being, is most hurtful, dangerous, and cruel; and no means will be found more efficacious in producing disease."*

I may here contradict the erroneous assertion, that the warm or tepid bath has a "weakening" effect on children. This is by no means true: rather by cleansing the pores of the skin, and thus assisting both perspiration and circulation, it is as beneficial to health as it is soothing and comfortable. I may add that, though mothers generally pay more attention to bathing children in the morning than at any other time, it is highly desirable that the child should be thoroughly cleansed from dirt and perspiration every evening before he is put to bed. The temperature of the bath may be *gradually* diminished, until, at the age of three or four months, and if the infant is healthy, the cold bath at 65° may be used every other morning instead of the tepid bath; but this must be discontinued if not followed by a glow of warmth.

During the use of the bath, particularly in cold weather, the air of the nursery should be comfortably warm, and the exposure of the naked body should not be unnecessarily protracted. Brisk but gentle friction of the body immediately on its removal from the water, and rapid dressing, will be the most likely means to secure the most beneficial effects of the bath. In this way most children may be gradually brought to the use of the *cold* bath every morning; but some will be found unable to bear it with impunity, however constantly it may have been employed, and it will be well for the mother to know when the use of the cold bath should be abandoned. If after the bath the child is chilly and languid, depressed in spirits, torpid and drowsy; if the countenance is pale, and the lips livid; and if the surface of the body continue cold, it is clear that the bath is injurious and should not be repeated. In cases where the cold bath cannot be borne, all the benefits of a general bath may frequently be obtained by means of a miniature shower bath. This may be done by dropping water on the head and shoulders, by means of a tin vessel perforated with holes, and held over it; a very convenient vessel may now be purchased for this purpose, the flow of water being regulated by the pressure of the thumb upon an opening in the handle.

Moderate exercise is advantageous before bathing; if the child is too young, friction of the body may be substituted.

The best period of *sea bathing* will be about two hours after breakfast: but the child must not be previously exhausted by over fatigue, nor should it afterwards be too

* Letters to a Mother. By J. T. Conquest, M.D.

much exposed to the cold currents of air on the sea shore.

Whatever form of bathing be adopted, all violent plungings should be avoided, as likely to excite the alarm of children, and to do more harm than good. The use of the bath should studiously be made as agreeable as possible, when it will soon be really enjoyed and anticipated ; but much here will obviously depend upon the judgment of the nurse.

LIGHT. If I were less confined to remarks immediately practical, I might raise here many very interesting questions respecting the influence of light upon health. Whatever may be its mode of action, the fact that it does act in a very important degree upon the animal economy is undoubted, though its influence is far less considered than that of air. When we observe the blanched countenances of children (especially such as reside in close murky lanes) in large towns, we are doubtless correct in ascribing such unyouthful symptoms in youth, partly to the want of fresh air and exercise : but we judge the case imperfectly if we do not take into consideration another serious want— *the want of a fair supply of natural light.*

If we require familiar instances of its influence, we have only to glance at some common facts in the vegetable world. Observe the effect which the gardener produces on the stem of the celery plant, simply by keeping it without light. If allowed to grow naturally, exposed to light, it would be strong, fibrous, and darkly coloured ; excluded from the light, it is as we see it on our tables, blanched and tender. I might also refer to the delicacy of structure, and the pallid hue which mark the species of fungi vegetating in the dark cellars.

Some time ago, a number of poor people went to reside in certain dark cells under the fortifications of Lisle, where they produced such a number of deformed infants, that it was deemed necessary to order the cells to be shut up, and not to be used as the habitations of human beings.

The practical lessons to be deduced from these facts are sufficiently obvious. They teach us freely to expose the infant to the light, as soon as the eyes have overcome their first extreme sensibility ; I speak of the diffused light of day, not of strong artificial light, for the latter is injurious. They teach us not to darken the nursery during the day, not to close the shutters during the night, on no account to use window curtains in the nursery, and, as often as is practicable, to remove the child from the murky atmosphere of the city, to the purer air and light of the suburbs. A full supply of light is essential in the *school-room.*

